# Antiviral Responses and Biological Concequences of *Piscine orthoreovirus* Infection in Salmonid Erythrocytes

**DOI:** 10.3389/fimmu.2018.03182

**Published:** 2019-01-16

**Authors:** Øystein Wessel, Aleksei Krasnov, Gerrit Timmerhaus, Espen Rimstad, Maria K. Dahle

**Affiliations:** ^1^Faculty of Veterinary Medicine, Norwegian University of Life Sciences, Oslo, Norway; ^2^Division of Aquaculture, Norwegian Institute of Fisheries and Aquaculture (Nofima), Tromsø, Norway; ^3^Department of Fish Health, Norwegian Veterinary Institute, Oslo, Norway; ^4^The Norwegian College of Fishery Science, UiT The Arctic University of Norway, Tromsø, Norway

**Keywords:** *Piscine orthoreovirus*, red blood cells, antiviral immunity, hemoglobin, salmonids, erythrocytes, anemia

## Abstract

Salmonid red blood cells are the main target cells for *Piscine orthoreovirus* (PRV). Three genotypes of PRV (PRV-1,2,3) infect Atlantic salmon (*Salmo salar*), Chinook salmon (*Onchorhynchus tshawytscha*), Coho salmon (*Oncorhynchus kisutch*), rainbow trout (*Onchorhynchus mykiss*) and brown trout (Salmo trutta), and can cause diseases like heart and skeletal muscle inflammation (HSMI), jaundice syndrome, erythrocyte inclusion body syndrome (EIBS) and proliferative darkening syndrome (PDS). Purified PRV administrated to fish has proven the causality for HSMI and EIBS. During the early peak phase of infection, salmonid erythrocytes are the main virus-replicating cells. In this initial phase, cytoplasmic inclusions called “virus factories” can be observed in the erythrocytes, and are the primary sites for the formation of new virus particles. The PRV-infected erythrocytes in Atlantic salmon mount a strong long-lasting innate antiviral response lasting for many weeks after the onset of infection. The antiviral response of Atlantic salmon erythrocytes involves upregulation of potential inhibitors of translation. In accordance with this, PRV-1 protein production in erythrocytes halts while virus RNA can persist for months. Furthermore, PRV infection in Coho salmon and rainbow trout are associated with anemia, and in Atlantic salmon lower hemoglobin levels are observed. Here we summarize and discuss the recently published findings on PRV infection, replication and effects on salmonid erythrocytes, and discuss how PRV can be a useful tool for the study of innate immune responses in erythrocytes, and help reveal novel immune functions of the red blood cells in fish.

## *Piscine Orthoreovirus* (PRV) Targets Salmonid Erythrocytes

The *Piscine orthoreovirus* (PRV) was first discovered in 2010 in Atlantic salmon (*Salmo salar*) suffering from the disease heart and skeletal muscle inflammation (HSMI) (1). Outbreaks of HSMI started to appear in Atlantic salmon aquaculture on the Norwegian west coast in 1999 (1, 2), occurring primarily a couple of months after transfer of salmon from fresh water facilities to net pens in the sea. The clinical signs were anorectic fish with abnormal swimming behavior, and accumulated mortality could be up to 20% of the population (3). The name of the disease, HSMI, was given due to the typical histological lesions; extensive heart inflammation starting with mononuclear infiltration of the epicardium which moves into the myocardium along with increased severity of the disease (3–5). Initial experimental trials showed transmission of HSMI to healthy fish after injection of heart homogenate, and a virus was suspected (6). However, it took ten more years before PRV was finally identified by RNA sequencing in 2010, in tune with the development in sequencing technology (1). *In silico* analyses of the viral genome defined PRV as the first *orthoreovirus* fully sequenced from fish, related to mammalian and avian orthoreoviruses (MRV, ARV) (7–10). PRV also has similarities to the grass carp reovirus (GCRV), which belong to the aquareoviruses. The compelling proof of causality between PRV and HSMI in Atlantic salmon was produced in 2017 when injected virus particles purified from fish blood were shown to transfer HSMI (11).

**Figure 1:**
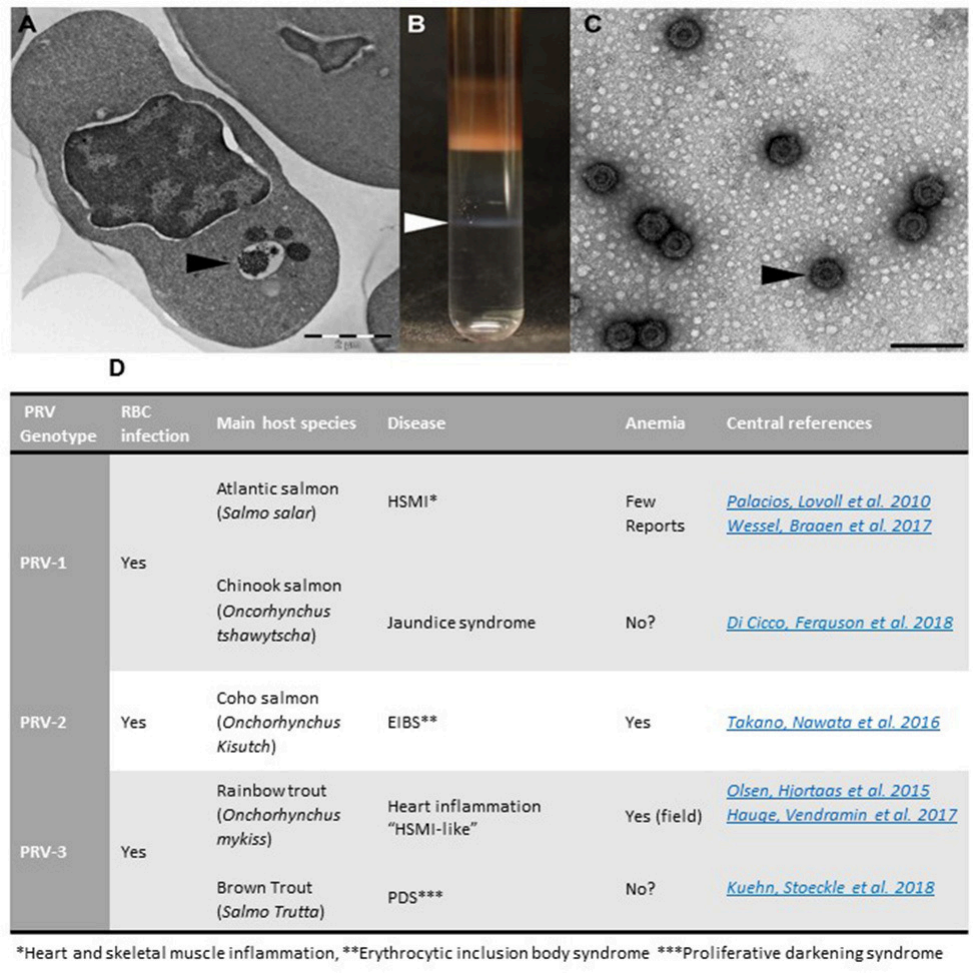
Purification of PRV from infected RBCs and PRV genotypes. **(A)** Micrograph of PRV infected erythrocyte with virus-containing inclusions in the cytoplasm (arrowhead). **(B)** Purification of PRV from infected blood cells results in virus band in CsCl gradient. **(C)** Electron microscopy image of purified PRV particles (Bar equals 100 nm). **(D)** Overview of PRV genotypes and associated diseases.

When antisera were developed to detect PRV *in situ* in sections from HSMI hearts, a surprising finding was made: the virus was not only present in cardiomyocytes, but also in unidentified blood cells (4). The infection of blood cells preceded the myocardial infection, and this was confirmed in experimental PRV infection (12). The findings of the latter study showed that the red blood cells (RBC), or erythrocytes, were the primary target cells for PRV in the primary phase of infection (12). Electron microscopy of the erythrocytes revealed cytoplasmic globular inclusions, which at the peak of infection were filled with reovirus-like particles (12) (Figure 1).

Orthoreoviruses have a segmented, double stranded RNA genome and replicate in the cellular cytoplasm. The virus particle, primarily based on studies of MRV, consists of eight structural proteins, whereas three non-structural proteins serve supportive functions related to the replication process in the infected cell (9). Orthoreoviruses have a double protein capsid with an inner core containing the genome, and an outer capsid with protruding surface proteins that can interact with cell surface receptors and glycans (13). Membrane interaction between orthoreoviruses and their cellular receptors trigger endocytosis, and in the endosome the outer reovirus capsid is partly digested, a process which exposes hydrophobic domains and triggers endosomal membrane penetration of the virus. Orthoreoviruses can also be subject to proteolysis in the extracellular environment, like the gut, into infectious subviral particles (ISVPs) which can cross the plasma membrane in a receptor-independent manner (14). In the cytoplasm the viral particle ends up as a stripped virus core containing the dsRNA genome, and the genome is transcribed and replicated by the virus' own RNA polymerase within the core. The resulting transcripts are further translated by the cellular translation system. A central protein is μNS, encoded by the virus segment M3. The μNS protein acts as a scaffold and bring the virus proteins together in the specific subcellular compartments (15–17). The PRV μNS protein will, when overexpressed in fish cells, form globular cytoplasmic clusters by itself, that resemble the clusters found in PRV-infected erythrocytes (18). PRV μNS directly interacts with several other PRV proteins, recruiting them to these clusters which are considered the production sites for viral progeny, so called “viral factories” (18). The virus factories increase in size but decrease in number during the virus cycle.

Fish red blood cells are nucleated and morphologically different from mammalian erythrocytes, with additional functions (19, 20). PRV infection in the red blood cells was discovered soon after first report on innate immune cell functions of rainbow trout erythrocytes, which were shown to respond to the dsRNA mimic poly(I:C) (19). The initial studies of PRV-infected erythrocytes revealed that the viral factories, consisting of PRV proteins and viral progeny, were also visible in a regular light microscope as dark spots in the red blood cells (12, 21). This visual image of dense cytoplasmic inclusions led to an assumption that PRV could also be responsible for a disease with a hitherto undefined etiology; the erythrocyte inclusion body syndrome (EIBS) (22, 23). EIBS had been described in wild and farmed salmonids in several countries as a disease with pathological characteristics different from HSMI, most strongly associated with anemia (23). A new PRV variant was identified in Coho salmon (*Onchorynchus kisutch*) suffering from EIBS, and purified virus was shown to form the inclusions typical for EIBS and anemia when given to fish experimentally (24). This PRV variant was named PRV-2.

To further link different pathological symptoms with PRV infection, another PRV genotype was detected in farmed rainbow trout (*Onchorhynchus mykiss*) in Norway, associated with both heart inflammation and anemia (25). Infection experiments performed with this PRV genotype, initially called PRV-*Om* (*Onchorhynchus mykiss*) in contrast to PRV-*Ss* (*Salmo salar*)/PRV-1, demonstrated formation of virus factory like structures in the cytoplasm of RBC. The rainbow trout PRV genotype is now referred to as PRV-3 (26).

PRV-*Om/*PRV-3 could infect both rainbow trout and Atlantic salmon, but preferably infected and caused disease in rainbow trout, whereas transmission and ability to cause disease in Atlantic salmon was negligible (27). In Chile, both PRV-*Ss*/PRV-1 and PRV-*Om/*PRV-3 was detected in rainbow trout (28).

The novel PRV genotypes have similar dissemination pattern and pathogenesis, but show preferences and differential pathogenicity for different salmonid species. The best established diseases are shown in table/Figure 1D. In addition, PRV-1 was recently associated with Jaundice syndrome in Chinook salmon (*Oncorhynchus tshawytscha*) (29); and PRV-3 with proliferative darkening syndrome (PDS) in Brown trout (*Salmo trutta)* (30).

PRV-1-3 all have erythrocytes as their main target cells in the initial peak phase of infection. The infected red blood cells contribute to the further virus dissemination to various host tissue, and the effects of PRV on erythrocytes in the different species may provide a key to an explanation of subsequent pathogenesis (11, 24, 27, 29).

## Effects of PRV Infection on Erythrocyte Gene Expression and Function

**Figure 2:**
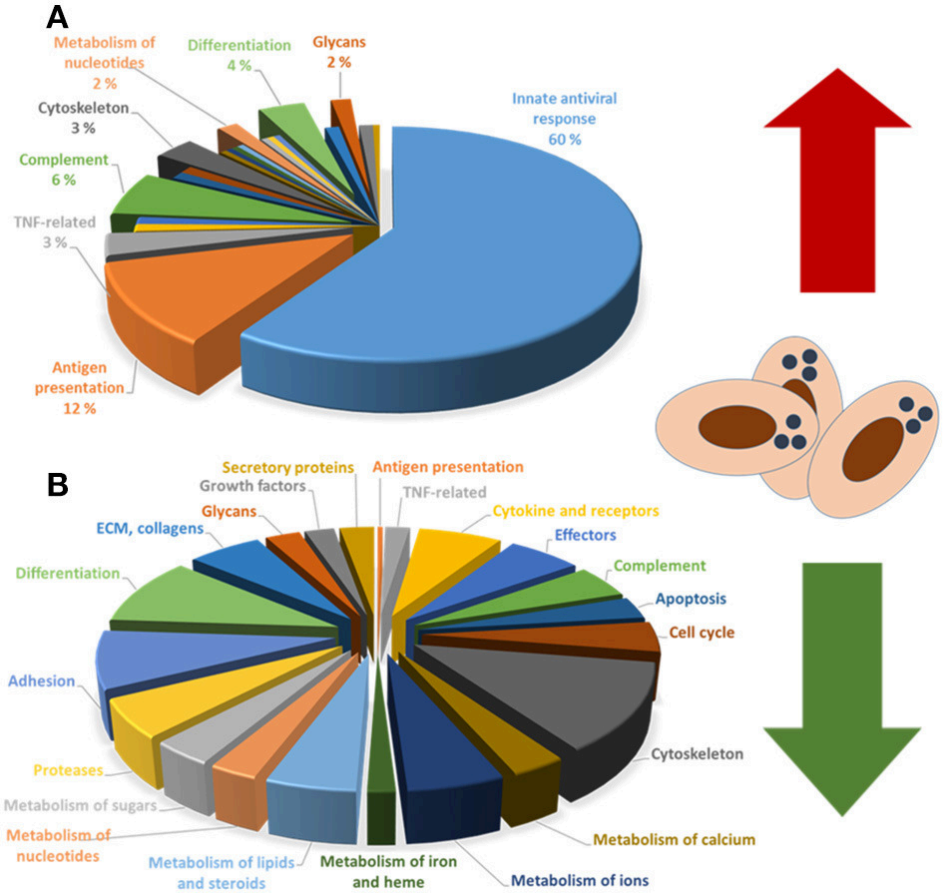
**(A)** Upregulated and **(B)** downregulated functional groups of genes in PRV-infected red blood cells.

The responses to PRV infection have been studied using DNA oligonucleotide microarray on red blood cells isolated from PRV-infected fish after experimental infection. These analyses revealed that that the infected erythrocytes strongly up-regulate a large group of genes associated with antiviral responses (31) (Figure 2A), similar to other tissues infected with RNA viruses in Atlantic salmon (32). The antiviral response correlated closely with increasing PRV levels in the red blood cells indicating that sensing and replication were linked in the early phase.

The main inducer of the antiviral response is cellular sensing of the PRV dsRNA genome. Two types of pattern recognition receptors (PRRs) are involved in dsRNA sensing in fish: the transmembrane dsRNA sensor toll-like receptor (TLR)3 in the endosomes and the cytoplasmic RIG-like receptors (RLRs) (33, 34). Trout red blood cells have been reported to express TLR3 and RIG-I (19), and induction by PRV was confirmed in red blood cells (19, 31). Fish cells also express TLR22 and TLR19, which are comparable to TLR3 by function, but is expressed primarily on the cell surface (35, 36). In mammals, intact MRV triggers both TLRs and RLRs, while intermediate subviral particles (ISVPs) formed by partial proteolysis of the outer viral capsid (e.g., in the gut) trigger RLRs only, leading to a less potent antiviral response (14). It is so far unknown if PRV infects RBCs as an intact particle or an ISVP.

The dsRNA receptors induce transcription of type I interferons through activation of interferon response factors (IRFs), which mediates further antiviral effects. Atlantic salmon RBCs express a panel of IRFs, of which expression of IRF7 showed highest correlation with PRV levels (31). In contrast to the mammalian IFNα and IFNβ, Atlantic salmon type I interferons form a large family divided into four groups IFNa–d, with several subtypes for each (37). In mammals, IFNβ is the main interferon induced by TLR3 and RLRs, and in line with this, MRV replication is reported to be controlled by IFNβ (38). Previous studies in fish have indicated that IFNa (1 and 2) serve similar functions as IFNβ in mammals (37, 39), which fits well with IFNa2 being one of the genes expressed with strongest correlation to PRV levels in Atlantic salmon RBC (31). Another study comparing expression of IFNa, b and c after PRV infection in a context of PRV cross-protection against IHNV, revealed preferential induction of IFNa (40). Secreted interferons bind to interferon receptors on surrounding cells, triggering a Jak/STAT signaling pathway leading to expression of multiple antiviral effector genes, which can inhibit further host dissemination of the virus. PRV-1 infection of Atlantic salmon RBCs induces numerous antiviral genes (31). Interestingly, among the induced genes correlating most strongly with PRV levels were the signaling mediators STAT1 and Jak1 themselves (17). The second largest gene group to be induced by PRV infection in RBC was genes involved in viral antigen presentation, including MHC class I antigen, tapasin and several proteosome subunits (31). This suggests that infected erythrocytes have the capability of presenting viral antigens to the immune system. A recent transcriptome/proteome study reported that rainbow trout RBC also express MHC Class II, (41). This points to salmonid erythrocytes as inducers of adaptive immunity. Another interesting finding in PRV-infected Atlantic salmon erythrocytes was that genes associated with immune suppression, like Interleukin 10 receptor and suppressor of cytokine signaling (SOCS) 1, was induced (31). SOCS1 is shown to suppress pathogen signaling and promote replication of salmonid alphavirus (SAV) in salmonid cell lines (42). Interestingly, PRV infection is cross-protective against SAV (43).

Responses of red blood cells have also been observed in Atlantic salmon infected with Piscine myocarditis virus (PMCV), a small dsRNA virus associated with cardiomyopathy syndrome (CMS) (44). Notably, PMCV is not shown to replicate in erythrocytes. This indicates that the RBC innate immune responses to viruses are induced independent of direct infection, which is also in line with reported responses to infectious pancreas necrosis virus (IPNV) (45).

Interestingly, we found that purified Atlantic salmon red blood cells infected with PRV in culture also induced IFNa in culture with subsequent induction of Mx and PKR, indicating that infected RBC produce functional IFNa (21). An interesting observation from RBC responses in culture, was the time course of PRV-mediated IFN and antiviral gene expression compared to the *in vivo* situation. In culture, IFNa expression peaked after 1 day and the antiviral effector genes after 1 week, followed by a decrease to basal levels after 2–3 weeks. The fact that the response did not decrease for months *in vivo* could point toward continuous infection of new RBCs, or to interferon stimulation from other sources.

Experimental infection of PRV-1 in Atlantic salmon and PRV-3 in rainbow trout has confirmed that PRV infects RBC prior to infection and induction of inflammatory lesions in the heart (11, 27). The same PRV-1 genotype have been associated with infection other target organs and differential disease pathology in Chinook salmon, where the disease is characterized by necrosis and degeneration in kidney and liver (29). Di Cicco et al proposed that a difference in PRV load tolerance in the RBC could be one reason for differential pathological outcomes from PRV-1 infection in the different species. Similarly, PRV-3 have been reported to infect both rainbow trout and Brown trout with differential outcomes (25, 30). Whereas, Heart inflammation and anemia was the most notable findings in farmed rainbow trout (25), wild brown trout suffered from proliferative darkening syndrome (PDS), a high mortality disease characterized by necrotic lesions and degeneration of the liver and to a lesser degree spleen and kidney (30). Hence, both PRV-1 and PRV-3 appear to cause species-specific diseases.

While PRV-3 appear to be cleared from blood after infection in rainbow trout (27), PRV-1 RNA persists in Atlantic salmon blood long after HSMI has healed and heart tissue has regenerated (11, 12, 46). The PRV-1 persistence can last for at least 50 weeks (47), which is in line with the >90% prevalence of PRV-1 detection in farmed Atlantic salmon. It is unknown if PRV-2 or PRV-3 can persist in a similar manner in their host species, but sustained carrier status has not been observed for these PRV genotypes so far (24, 27).

When targeting PRV proteins with antibodies instead of analyzing PRV RNA, a quite different result is obtained. PRV protein production peaks for a couple of weeks and is then decreased to undetectable levels (11, 12, 46). This discrepancy between PRV RNA- and protein levels points to translational blocking or degradation of viral protein. In line with this, the protein decrease coincides with the peak gene expression of the antiviral effectors (46).

When comparing with the mammalian counterpart, MRV has been reported to counteract the antiviral response in several ways to support its own replication. Mechanisms of interaction include binding and inactivation of IRF3 by the PRV μNS protein (14), or bypassing translational blocking through the PRV σ3 protein (48). The long lasting transcription of interferon-regulated genes in PRV-infected A. salmon indicates that PRV does not effectively block interferon production or interferon-mediated stimulation of antiviral gene expression in Atlantic salmon RBC. However, there may be a block at the translational level. The persistence of PRV RNA along with an apparent block in progeny production points toward ineffective eradication of infected RBC, while viral dissemination appears to be held back by innate immune mechanisms.

When comparing antiviral responses to PRV-1 and PRV-3 cross-species, data so far indicate that the magnitude of innate antiviral responses corresponds to virulence in the respective target species (PRV-1 in A. salmon and PRV-3 in rainbow trout). Although PRV-1 can replicate intensely in Coho salmon and Sockeye salmon (*O. nerka*) blood cells, the antiviral response to PRV-1 in Sockeye salmon is reported to be very low (49). Similarly, PRV-3 replicates in Atlantic salmon blood but induce weak antiviral response. This implies that the pathologic effects of PRV infection could be coupled to the ability to induce antiviral immune responses in the host.

Anemia is reported as a hallmark for PRV-2 infected Coho salmon, and typical for PRV-3 infected farmed rainbow trout (24, 25). In an infection that can affect up to 50% of the erythrocytes in the peak phase, anemia is not unexpected. However, experimental studies of PRV-3 infected rainbow trout have not reproduced the anemia observed in field outbreaks (12, 27). Similarly, anemia is not produced in PRV-1 experimental trials (11), and not commonly observed in farmed PRV-1 infected A. salmon in Norway. In contrast, reports from Chile have indicated that 19% of HSMI diseased fish had pale gills and heart, which could indicate anemia (50). Genes associated with erythropoiesis are found to be induced in spleen after PRV infection in Atlantic salmon (51), indicating that cleared, erythrocytes are replaced efficiently enough to avoid anemia. However, a reduction in hemoglobin is observed in PRV-infected RBC in the period after the peak virus production in blood (11, 52). The hemoglobin reduction is observed after the peak in virus levels, suggesting that it may be caused by the same translational block that reduces production of viral progeny (11, 46). A direct effect of PRV-1 infection on the ability of Atlantic salmon to tolerate hypoxia was revealed in a stress test experiment, which indicated that PRV-infected fish could be more prone to mortality due to stress or crowding at suboptimal oxygen conditions (52), possibly due to hemoglobin reduction.

The transcriptome study of PRV-1 infected RBCs indicated a general decrease in the expression of a range of functional gene groups. Although not fully understood in relation to implications, the expression patterns strongly indicated that PRV infection repressed genes that controlled erythrocyte shape/cytoskeleton, tissue interaction/adhesion, cell-cell communication/cytokines/chemokines and metabolism (31) (Figure 2B). This effect could be caused by infection and antiviral immunity. In addition, adrenergic stress responses are reported to reduce transcript stability in fish red blood cells (53), and could be partly responsible for this effect.

Among the genes less suppressed by the infection were genes directly related to heme and hemoglobin synthesis, which supports the hypothesis that the hemoglobin reduction associated with EIBS and HSMI is primarily due to post trancriptional effects. Strong reduction in adhesion molecule expression implies that the ability of RBC to interact with muscle tissue for oxygen delivery could be affected, and thereby add to the physiological consequences of hemoglobin reduction. When keeping in mind that a translational inhibition may further add to suppression at the transcriptional level, disturbed gas exchange to muscle tissue would not be a surprising result. Clarifying if these findings are coupled to PRV-mediated disease will be an important step forward. In addition, the study of PRV infection is a key to understanding the immunological role of fish erythrocytes.

## Author Contributions

ØW wrote about PRV virus, Figure 1, read and approved the manuscript. AK wrote about transcriptome data, Figure 2, read and approved the manuscript. GT wrote about transcriptome data, read and approved the manuscript. ER wrote about PRV virus, read and approved the manuscript. MD coordinated the draft, wrote about disease, immunology and erythrocyte functions, read and approved the manuscript.

### Conflict of Interest Statement

The authors declare that the research was conducted in the absence of any commercial or financial relationships that could be construed as a potential conflict of interest. The handling editor is currently co-editing a Research Topic with one of the authors ER, and confirms the absence of any other collaboration.
